# The inhibitory impact of various total body irradiation doses on the hematopoietic system of mice

**DOI:** 10.1097/BS9.0000000000000214

**Published:** 2024-12-20

**Authors:** Hui Xu, Jinwang Zhang, Hexiao Zhang, Ming Yang, Wenshan Zhang, Wei Wang, Chaoqun Wang, Yiran Zhang, Zhongxiang Jiao, Yingdai Gao, Yinghui Li

**Affiliations:** a State Key Laboratory of Experimental Hematology, National Clinical Research Center for Blood Diseases, Haihe Laboratory of Cell Ecosystem, PUMC Department of Stem Cell and Regenerative Medicine, CAMS Key Laboratory of Gene Therapy for Blood Diseases, Institute of Hematology and Blood Diseases Hospital, Chinese Academy of Medical Sciences & Peking Union Medical College, Tianjin 300020, China; b Tianjin Institutes of Health Science, Tianjin 301600, China; c Tianjin Key Laboratory of Biomaterial Research, Institute of Biomedical Engineering, Chinese Academy of Medical Sciences and Peking Union Medical College, Tianjin 300192, China

**Keywords:** Extramedullary hematopoiesis, Colony-forming, Irradiation toxicity, Mouse transplantation

## Abstract

Irradiation with X-rays has been widely utilized in the clinical treatment of solid tumors and certain hematopoietic malignancies. However, this method fails to completely distinguish between malignant and normal cells. Prolonged or repeated exposure to radiation, whether due to occupational hazards or therapeutical interventions, can cause damage to normal tissues, particularly impacting the hematopoietic system. Therefore, it is important to investigate the effects of total body irradiation on the hematopoietic system of mice and to compare the inhibitory effects of various doses of irradiation on this system. In this study, we primarily employed flow cytometry to analyze mature lineage cells in the peripheral blood, as well as immature hematopoietic stem and progenitor cells (HSPCs) in the bone marrow and spleen. Additionally, we evaluated the multilineage differentiation capacity of HSPCs through colony-forming cell assays. Our results indicated that peripheral B and T cells demonstrated increased sensitivity to irradiation, with significant cell death observed 1-day post-irradiation. Common lymphoid progenitor cells exhibited greater radiotolerance compared to other progenitor cell types, enabling them to maintain a certain population even at elevated doses. Moreover, notable differences were observed between intramedullary and extramedullary hematopoietic stem cells and common lymphoid progenitor cells regarding the extent of damage and recovery rate following irradiation. The multilineage differentiation capacity of HSPCs was also compromised during radiation exposure. In conclusion, different types of mature blood cells, along with immature HSPCs, exhibited varying degrees of sensitivity and tolerance to irradiation, resulting in distinct alterations in cell percentages and numbers.

## 1. INTRODUCTION

Irradiation with X-rays induces the generation of free radicals, particularly reactive oxygen species, which can lead to DNA damage and subsequent cell necrosis.^1,2^ Based on this principle, radiotherapy has been extensively utilized in clinical settings to eliminate and inhibit the growth of various malignant solid tumors since 1896, when the first successful clinical application of irradiation was performed.^3^ However, irradiation cannot completely differentiate between malignant and normal cells, often resulting in toxicity to normal tissues and leading to numerous adverse reactions.^4^ Furthermore, prolonged or repeated exposure to radiation, whether due to occupational hazards or radiotherapy, can cause significant damage to the hematopoietic system and may increase the risk of developing cancers, including hematopoietic malignancies.^5–8^ Therefore, it is important to analyze the effects of irradiation on various types of mature cells, as well as on hematopoietic stem and progenitor cells (HSPCs), and to investigate how these effects change over time.

Hematopoietic stem cells (HSCs) are the foundational cells in the body responsible for generating all myeloid and lymphoid blood cells, thereby ensuring a continuous supply of blood through a highly organized hematopoietic hierarchy.^9^ In this hierarchy, multipotent stem cells differentiate into oligopotent progenitor cells, which subsequently develop into lineage-committed effector cells, ultimately giving rise to a diverse array of mature functional cells.^10^ High-dose total body irradiation (TBI) has been widely employed as part of the conditioning regimen prior to hematopoietic cell transplantation (HCT) for patients with hematopoietic malignancies and is aimed at eliminating residual malignant cells and host immune cells while ablating bone marrow (BM) hematopoiesis to create space for the transplanted HSCs.^11^ However, due to the significant toxicity associated with high-dose irradiation, reduced-intensity conditioning regimens have gradually emerged as the predominant pretreatment strategy.^12,13^ Although the irradiation dose is primarily determined by clinical practice, it is fundamentally influenced by the varying sensitivities of distinct hematopoietic cells to irradiation. Therefore, ensuring the thoroughness of myeloablation necessitates exceeding the maximum resistance of these key cells to irradiation. Consequently, it is essential to investigate the sensitivity and resistance of different hematopoietic cells to irradiation.

In this study, we investigated the responses of mature differentiated cells in the periphery, as well as HSCs and various progenitor cells within both intramedullary and extramedullary hematopoietic tissues, specifically the BM and spleen of mice, to exposure to varying doses of irradiation. Additionally, we examined how these effects change over time. A comprehensive understanding of the sensitivity of various hematopoietic cells to irradiation, as well as their recovery over time following exposure, is highly valuable for guiding the treatment of blood damage resulting from irradiation.

## 2. MATERIALS AND METHODS

### 2.1. Animals

Female C57BL/6J mice (CD45.2^+^, 6–7 weeks old) were purchased from Beijing HFK Bioscience Co., Ltd., (Beijing, China) and maintained under specific pathogen-free (SPF) conditions. They were housed in a 12-hour light/dark cycle, a temperature range of 18°C to 23°C, and a humidity level of 40% to 60%, with free access to food and water. Animal experimental protocols received approval NO. IHCAMS-DWLL-NSFC2024010-1 from the Animal Care and Use Committee of State Key Laboratory of Experimental Hematology, Institute of Hematology and Blood Diseases Hospital.

### 2.2. TBI treatment

After 1 week of adaptive feeding, mice were randomly divided into 6 equally sized experimental groups based on their bodyweight (18.51 ± 0.61 g). These groups were administered TBI in an increasing dose regimen of 0 (control), 2, 4, 6, 8, and 10 Gy, delivered at a dose rate of 0.615 Gy/min using X-rays (RS2000 Pro, Rad source). Evaluations were conducted at 4 hours, 1 day, 4 days, 7 days, 10 days, and 13 days post-irradiation. Both the 8 and 10 Gy groups received fractional irradiation in 2 equal doses, separated by an interval of 4 hours.

### 2.3. Cell preparation

To study the bone marrow nucleated cells (BMNCs), the mice were euthanized via carbon dioxide asphyxiation and subsequently immersed in 75% alcohol. BM cells were extracted from the ilia, femurs, and tibias using a 23-gauge needle, and were flushed into 1× phosphate-buffered saline (PBS) containing 2% fetal bovine serum (FBS, Gibco), referred to as PBS (2% FBS). The cell suspension was then passed through a 400-mesh filter to achieve a single-cell suspension. To remove mature red blood cells, red blood cell lysis buffer (eBioscience) was utilized, allowing for the isolation of BMNCs. The BMNCs from each mouse were counted using an automatic cell counter and subsequently stored at 4°C for further experiments.

For the isolation of splenic nucleated cells (SPNCs), mice were euthanized via carbon dioxide asphyxiation and subsequently immersed in 75% alcohol. The spleens were then homogenized in PBS (2% FBS) and filtered through a 400-mesh filter. Following this, the remaining red blood cells were lysed and removed to isolate the SPNCs. The SPNCs from each mouse were counted and stored at 4°C until required for experiments.

To obtain peripheral blood nucleated cells (PBNCs), 10 to 15 µL of peripheral blood was drawn from living mice by clipping the tail tip. Mature red blood cells were then lysed and removed to isolate PBNCs, which were also stored at 4°C for subsequent experiments.

### 2.4. Flow cytometry analysis

PBNCs were stained with fluorophore-conjugated anti-mouse antibodies as follows: PerCP-Cy5.5-labeled CD45.2 (BD, 552950), APC-Cy7-labeled CD3 (Biolegend, 100330), FITC-labeled CD19 (Biolegend, 152404), PE-Cy7-labeled NK1.1 (Invitrogen, 25-5941-82), APC-labeled CD11b (Invitrogen, 17-0112-81), and BV510-labeled Gr-1 (Biolegend, 108457) in PBS (2% FBS) at 4°C for 30 minutes. Following this, the stained cells were washed once with PBS (2% FBS). Cell doublets and dead cells were excluded using 4', 6-diamidino-2-phenylindole (DAPI) staining. T cells were identified as CD45.2^+^CD3^+^, B cells were identified as CD45.2^+^CD19^+^, natural killer (NK) cells were identified as CD45.2^+^CD3^−^NK1.1^+^, natural killer T (NKT) cells were identified as CD45.2^+^CD3^+^NK1.1^+^, monocytes were identified as CD45.2^+^CD11b^+^Gr-1^−^, and neutrophils were identified as CD45.2^+^CD11b^+^Gr-1^+^.

Subsequently, we took 1 × 10^6^ BMNCs or SPNCs per sample and stained them in PBS (2% FBS) with the following fluorophore-conjugated anti-mouse antibodies: PerCP-Cy5.5-labeled CD45.2 (BD, 552950), PE-Cy7-labeled Lin (Invitrogen), APC-labeled c-kit (Invitrogen, 17-1171-83), BV786-labeled sca-1 (BD, 563991), BV605-labeled CD34 (BD, 750918), BV711-labeled CD16/32 (BD, 751691), PE-CF594-labeled Flt3 (BD, 562537), APC-Cy7-labeled CD127 (Biolegend, 135040), FITC-labeled CD41 (BD, 561849), and AF700-labeled CD71 (Invitrogen, 56-0711-82) at 4°C for 30 minutes. Following this, the stained cells were washed once with PBS (2% FBS). After excluding cell doublets and dead cells using DAPI, HSCs were identified as Lin^−^sca-1^+^c-kit^+^; common myeloid progenitors (CMPs) were identified as Lin^−^sca-1^−^c-kit^+^CD34^+^CD16/32^−^; megakaryocyte-erythroid progenitors (MEPs) were identified as Lin^−^sca-1^−^c-kit^+^CD34^−^CD16/32^−^; granulocyte-monocyte progenitors (GMPs) were identified as Lin^−^sca-1^−^c-kit^+^CD34^+^CD16/32^+^; and common lymphoid progenitors (CLPs) were identified as Lin^−^sca-1^lo^c-kit^lo^CD127^+^ Flt3 (CD135)^+^. The Lin marker includes antibodies Ter119 (25-5921-82), Gr-1 (25-5931-82), CD11b (552850), B220 (25-0452-82), CD3 (25-0031-82), CD4 (25-0041-82), and CD8 (25-0081-82).

### 2.5. Colony-forming cell (CFU) assay

The mouse BMNCs and SPNCs were adjusted to 50 µL per 100,000 cells and 50 µL per 1000,000 cells, respectively, in Iscove Modified Dulbecco Medium (IMDM) containing 2% FBS. The frequencies of CFUs were estimated by plating 10 µL of the cell suspension in 1 mL of MethoCult™ GF M3434 (StemCell Technologies, Canada) in 6-well plates (Corning). After 10 to 12 days in culture, the plates were visually scored for colony-forming unit-granulocyte/macrophage (CFU-GM), colony-forming unit-erythrocyte (CFU-E), burst-forming unit-erythroid (BFU-E), and colony-forming unit-granulocyte/erythrocyte/macrophage/megakaryocyte (CFU-GEMM).

### 2.6. Complete blood count

Approximately 15 µL of peripheral blood was collected from living mice by clipping the tail tip and was subsequently suspended in 105 µL PBS containing 2 mM ethylenediaminetetraacetic acid (EDTA; Sigma-Aldrich). These samples were then analyzed using a pre-dilution model of an automated hematology analyzer (Sysmex) to determine the count and morphology of peripheral blood cells.

### 2.7. Statistical analysis

All data were presented as the mean ± standard deviation (SD) and all statistical analyses were conducted using GraphPad Prism version 8.4.0 (GraphPad Software). Two-tailed Student *t* tests were employed to calculate *P* values for the majority of the data sets, with *P* values <.05 considered indicative of statistically significant results. The significance levels are denoted as follows: *P < .05, **P < .01, ***P < .001, ****P < .0001, and ns: not significant.

## 3. RESULTS

### 3.1. TBI affects bodyweight and survival of mice

**Figure 1.**
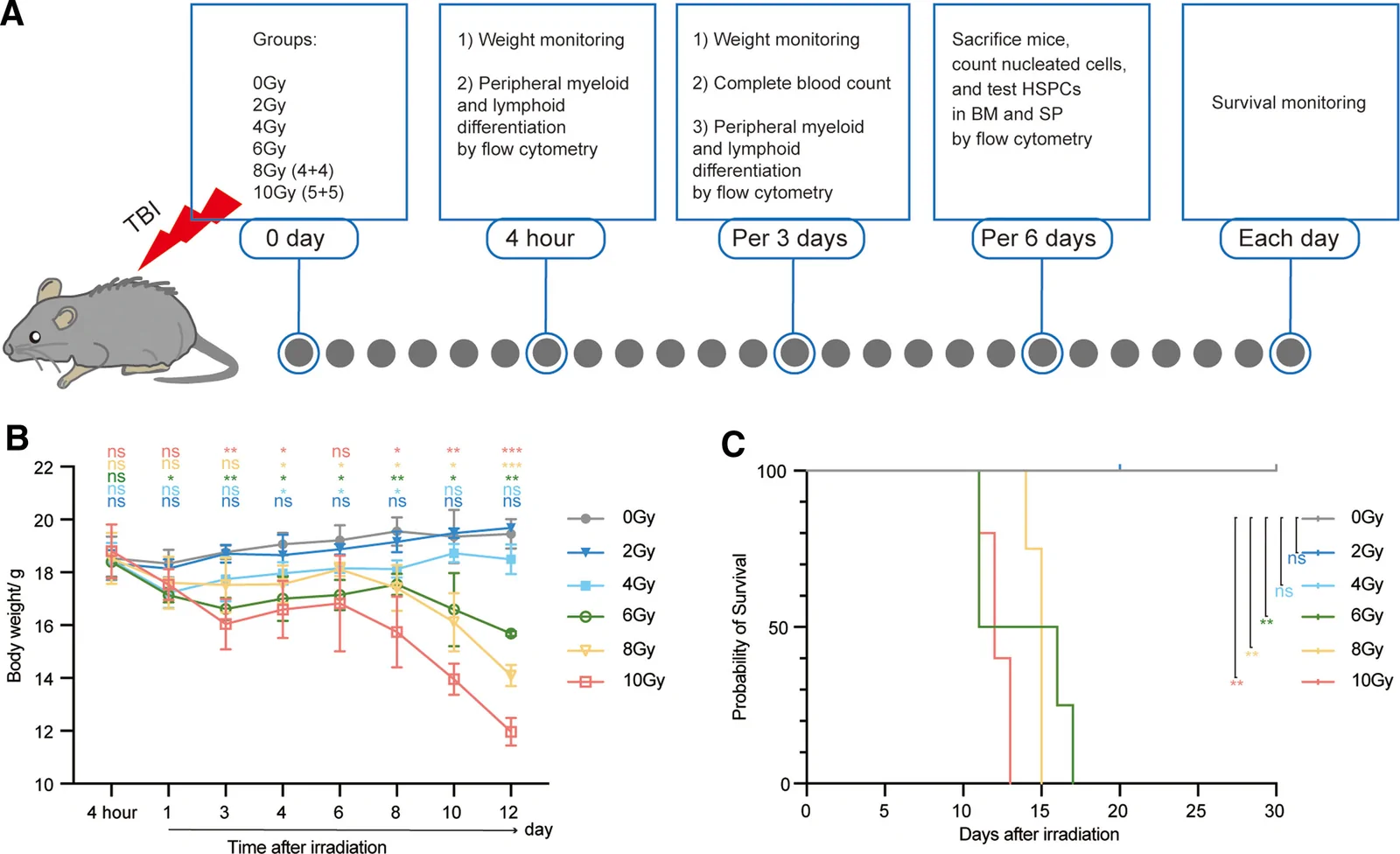
TBI caused reduced bodyweight and shortened survival time in mice. (A) Schematic diagram of the experiment. (B) Body weight measurements of mice post-irradiation (n = 3). Differences were calculated by comparing each dose group to the non-irradiated group at each time point. (C) Kaplan–Meier survival curves for mice subjected to different irradiation dosage groups (n = 4–6). Survival differences among the groups were analyzed using the Kaplan–Meier log-rank test in a pairwise manner. BM = bone marrow, HSPC = hematopoietic stem and progenitor cell, SP = spleen, TBI = total body irradiation.

Healthy adult mice were divided into 6 groups and subjected to TBI at increasing doses of 0, 2, 4, 6, 8, or 10 Gy (**Fig. 1A**). The overall health of the mice was monitored and evaluated through measurements of bodyweight and survival following irradiation. In the groups exposed to lower doses (0 and 2 Gy), bodyweight steadily increased post-irradiation (**Fig. 1B**). In contrast, at higher doses, the 4 Gy group initially exhibited a slight decrease in bodyweight 1-day post-irradiation, which subsequently stabilized (**Fig. 1B**). However, the 6, 8, and 10 Gy groups experienced a dramatic and prolonged decline in bodyweight, failing to return to baseline levels (**Fig. 1B**). Additionally, the mice that received doses greater than 6 Gy died within 11 to 17 days following irradiation (**Fig. 1C**). These data indicate that high-dose irradiation inflicts irreversible damage to mouse bodyweight and survival, consistent with findings from a previous study.^14^

### 3.2. TBI disrupts the homeostasis of peripheral blood cells of mice

**Figure 2.**
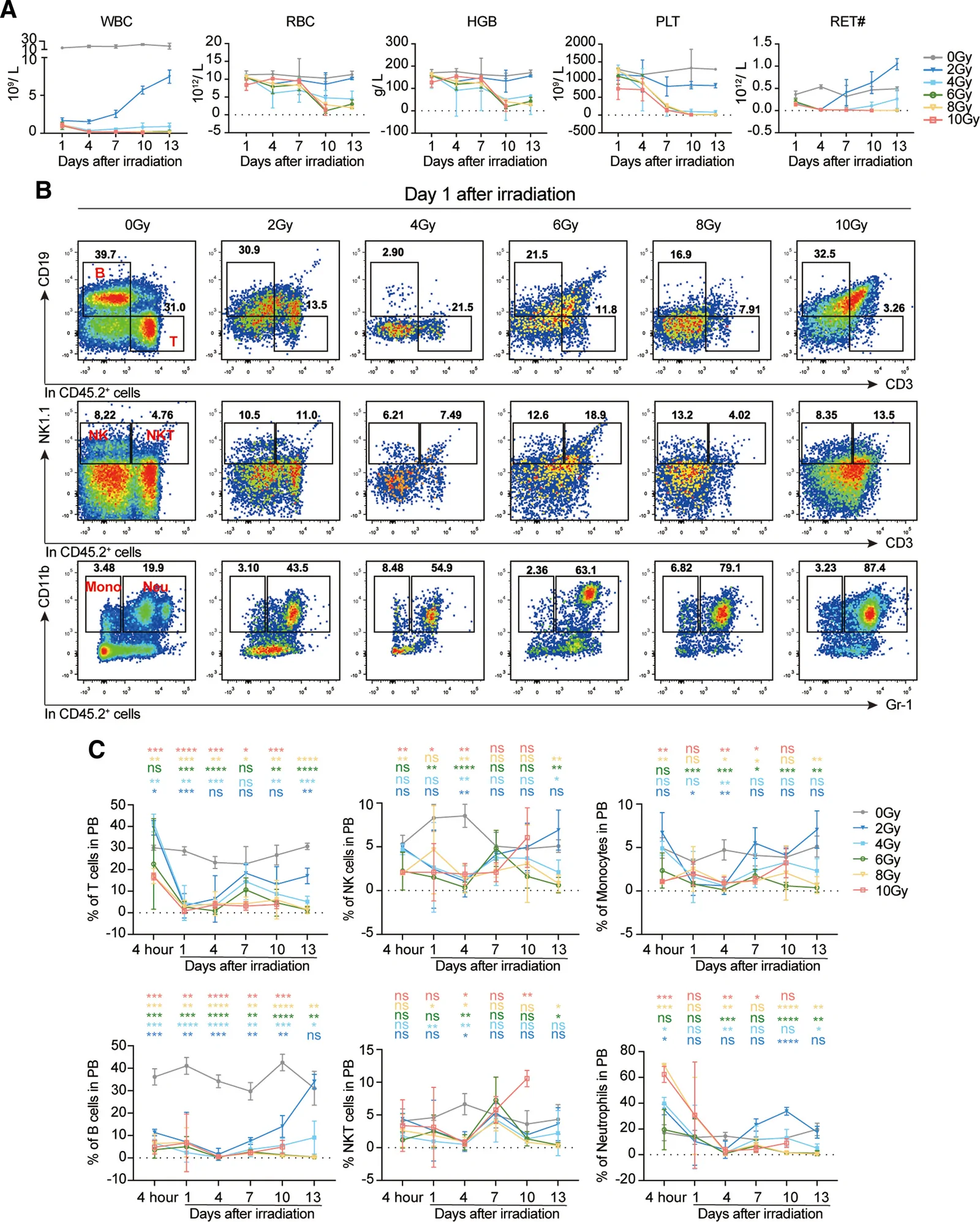
Irradiated mice exhibited peripheral disruption. (A) Complete blood count analysis of PB, WBC, PLT, RBC, HGB, RET in irradiated mice (n = 3). (B) Representative profiles and (C) the percentage of T cells, B cells, NK cells, NKT cells, monocytes, and neutrophils in the PB of irradiated mice after irradiation (n = 3) at day 13 after irradiation. Differences were calculated by comparing each dose group to the non-irradiated group at each time point. HGB = hemoglobin, NK = natural killer, NKT = natural killer T, PB = peripheral blood, PLT = platelets, RBC = red blood cells, RET = reticulocytes, WBC = white blood cells.

Peripheral blood cells (PBCs) comprise mature, differentiated myeloid and lymphoid cells derived from HSCs, playing crucial roles in oxygen and nutrient supply, as well as in the defense against pathogens.^15^ Impaired hematopoiesis can result in an abnormal supply of PBCs, and any abnormal death or reduction in these cells can trigger increased hematopoiesis. Consequently, peripheral blood serves as a biological sample that is rich in information, easy to obtain, and less harmful to mice compared to samples from other sources. In this study, we examined the changes in peripheral blood components in mice at 4 hours, 1 day, 4 days, 7 days, 10 days, and 13 days following irradiation (**Fig. 1A**). Complete blood count measurements revealed that the number of white blood cells (WBC) declined to a minimum 1 day after irradiation (**Fig. 2A**), with no significant differences observed between the irradiated groups (data not shown). Similarly, the levels of red blood cells (RBC), hemoglobin (HGB), platelets (PLT), and reticulocytes (RET) gradually decreased during the first 7 days post-irradiation, followed by a sharp decline thereafter, failing to return to normal values with higher irradiation doses (**Fig. 2A**). Given the distinctive behavior of WBC counts in response to irradiation, we utilized flow cytometry to further investigate the changes in primary lymphocytes following exposure.

We gated the CD45.2^+^ WBCs in live cells to analyze the changes in various lymphocyte populations following irradiation (**Fig. 2B**). Unlike the percentages of NK cells, NKT cells, monocytes, and neutrophils, T cells and B cells exhibited more regular fluctuations (**Fig. 2C**). Four hours post-irradiation, the percentages of B cells in the irradiated groups decreased sharply (**Fig. 2C**), indicating that B cells are particularly sensitive and susceptible to irradiation damage. The percentage of T cells in the high-dose (≥6 Gy) irradiated groups also significantly decreased; however, T cells in the low-dose irradiated groups experienced a notable short-term increase 4 hours post-irradiation (**Fig. 2C**), suggesting a unique transient response of T cells to external stimuli. Nevertheless, the transient proliferation did not mitigate the cell damage caused by high-dose irradiation. T cells from all irradiated groups decreased to their first minimum 1 day post-irradiation (**Fig. 2C**). While other lymphocyte populations did not reach their first minimum until day 4 after irradiation (**Fig. 2C**). Only the recovery of T and B cells exhibited more regular fluctuations, with higher irradiation doses hindering the recovery of these immune cells (**Fig. 2C**). Collectively, these data illustrate that different lymphocyte types exhibit varying levels of tolerance and resilience to irradiation, with high-dose irradiation tending to induce irreversible damage to PBCs.

### 3.3. TBI causes damage to HSPCs in the BM and spleen

**Figure 3.**
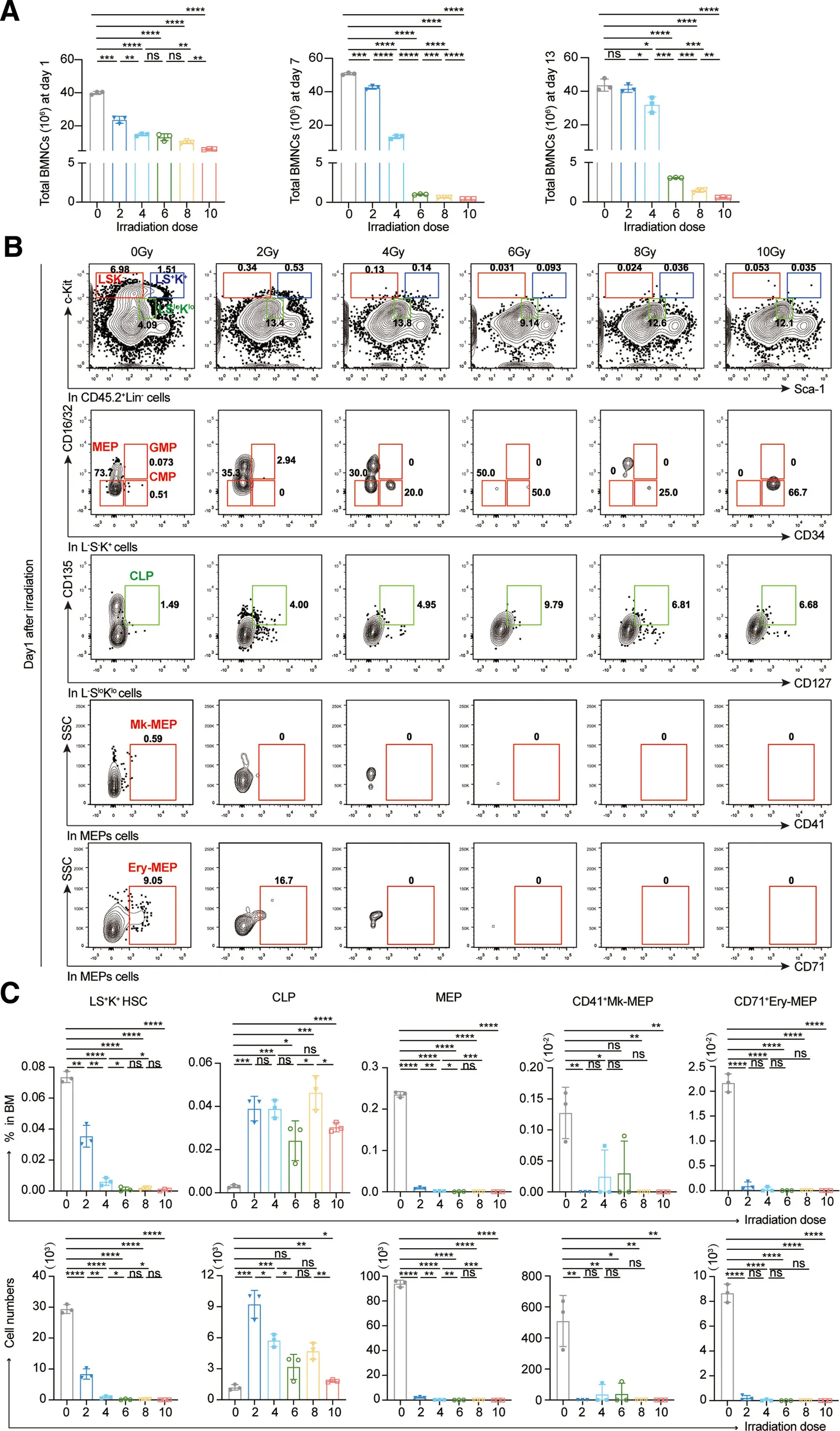
Irradiation caused significant damage to the bone marrow hematopoietic stem and progenitor cells 1 d post-irradiation. (A) Total cell numbers of BMNCs at day 1, 7, and 13 post-irradiation (n = 3). (B) Representative profiles and (C) the percentage (upper) and absolute cell numbers (lower) of various HSPC subsets, including Lin^−^Sca-1^+^c-Kit^+^ (LS^+^K^+^) HSCs, LS^lo^K^lo^ CD127^+^CD135^+^ CLPs, LS^−^K^+^ CD34^−^CD16/32^−^ MEPs, CD41^+^ Mk-biased MEPs, and CD71^+^ Ery-biased MEPs at day 1 post-irradiation. BMNC = bone marrow nucleated cells, CLP = common lymphoid progenitors, CMP = common myeloid progenitor, Ery = erythrocyte, GMP = granulocyte-monocyte progenitor, HSC = hematopoietic stem cell, HSPC = hematopoietic stem and progenitor cell, MEP = megakaryocyte-erythroid progenitors, Mk = megakaryocyte, SSC = side scatter.

Various mature PBCs are terminally differentiated cells derived from HSPCs. Many of these cells primarily reside in the BM, while others are found in the extramedullary hematopoietic tissues, such as the spleen.^16,17^ Consequently, we investigated the effects of TBI on HSPCs within both the BM and spleen, as these effects inevitably influence the recovery of PBCs following irradiation. Initially, we observed that the quantities of BMNCs and SPNCs exhibited a dose-dependent decline 1-day post-irradiation (**Fig. 3A** and Supplementary Fig. 1A, http://links.lww.com/BS/A106). One day after irradiation, the number of most progenitor cells, including LS^−^K^+^CD34^+^CD16/32^−^ CMPs, LS^−^K^+^CD34^+^CD16/32^+^ GMPs, LS^−^K^+^CD34^−^CD16/32^−^ MEPs, CD41^+^ megakaryocyte (Mk)-biased MEPs, and CD71^+^ erythrocyte (Ery)-biased MEPs, rapidly decreased to nearly zero across all treatment groups, indicating a reduced resistance to irradiation (**Fig. 3B, C** and Supplementary Fig. 1B, C, http://links.lww.com/BS/A106). The number of LS^lo^K^lo^CD127^+^CD135^+^ CLPs was significantly higher in the BM of all treatment groups compared to the 0 Gy group (**Fig. 3B, C**), whereas splenic CLPs were markedly lower than those in the 0 Gy group (Supplementary Fig. 1B, C, http://links.lww.com/BS/A106). This suggests that BM CLPs, rather than splenic CLPs, increased following irradiation. Furthermore, the reduction in the number of Lin^−^Sca-1^+^c-Kit^+^ (LS^+^K^+^) HSCs in the spleen after irradiation was significantly greater than that observed in the BM, indicating that the impact of irradiation on splenic HSCs was more severe than on BM HSCs (**Fig. 3C** and Supplementary Fig. 1C, http://links.lww.com/BS/A106).

**Figure 4.**
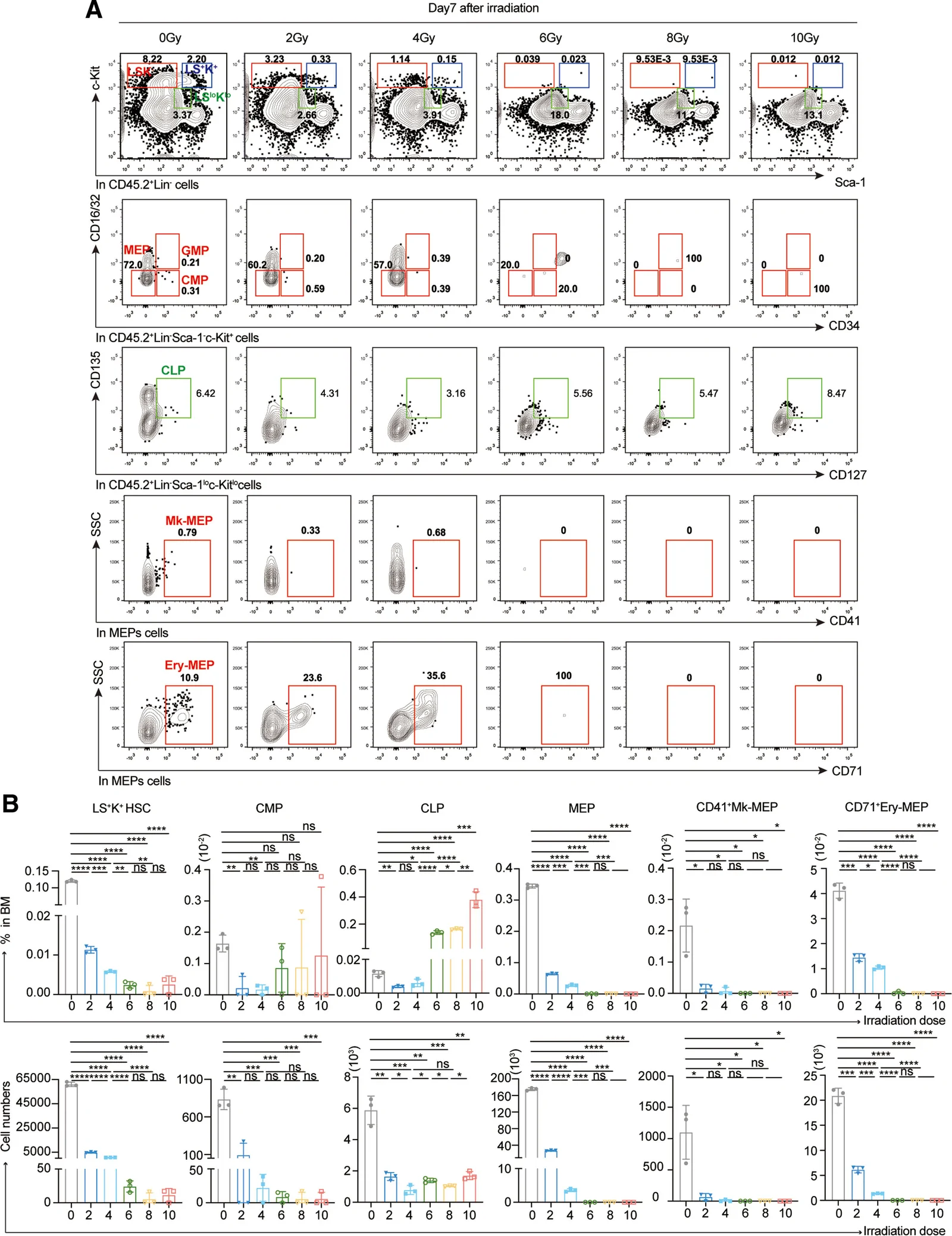
The irradiation damage to hematopoietic stem and progenitor cells in the bone marrow reached its lowest level at day 7 post-irradiation. (A) Representative profiles and (B) the percentage (upper) and absolute cell numbers (lower) of various hematopoietic stem and progenitor cell subsets, including Lin^−^Sca-1^+^c-Kit^+^ (LS^+^K^+^) HSCs, LS^−^K^+^CD34^+^CD16/32^−^ CMPs, LS^lo^K^lo^CD127^+^CD135^+^ CLPs, LS^−^K^+^CD34^−^CD16/32^−^ MEPs, CD41^+^ Mk-biased MEPs, and CD71^+^ Ery-biased MEPs at 7 d following irradiation (n = 3). BM = bone marrow, CLP = common lymphoid progenitors, CMP = common myeloid progenitors, Ery = erythrocyte, HSC = hematopoietic stem cell, MEP = megakaryocyte-erythroid progenitors, Mk = megakaryocyte, SSC = side scatter.

By day 7 following irradiation, the counts of BMNCs and SPNCs in the 4 to 10 Gy groups continued to decrease, reaching their lowest levels (**Fig. 3A** and Supplementary Fig. 1A, http://links.lww.com/BS/A106). In contrast, the count in the 2 Gy group had begun to increase, although it had not yet returned to normal levels (**Fig. 3A** and Supplementary Fig. 1A, http://links.lww.com/BS/A106). Figure 4B demonstrates that TBI at a dose of 2 Gy resulted in a dramatic reduction in the number of HSCs in the BM and spleen (92.1% vs 78.9%, respectively), with further declines observed as the irradiation dose increased (**Fig. 4B** and Supplementary Fig. 2B, http://links.lww.com/BS/A106). Similarly, GMPs, MEPs, Mk-MEPs, and Ery-MEPs exhibited persistently low levels in both the BM and spleen (**Fig. 4C** and Supplementary Fig. 2B, http://links.lww.com/BS/A106). Notably, at lower exposure doses (2 or 4 Gy), the percentages of CLPs were significantly lower than those in the 0 Gy group, while they dramatically increased with higher irradiation doses (≥6 Gy), leading to relatively high numbers of CLPs in both the BM and spleen (**Fig. 4B** and Supplementary Fig. 2B, http://links.lww.com/BS/A106). Furthermore, compared to the number at day 1 post-irradiation, BM CLPs at day 7 post-irradiation were significantly lower than those in the 0 Gy group. This indicates that the rapid increase in BM CLPs following irradiation was temporary, which is an important finding, as an adequate number of CLPs is essential for the replenishment of peripheral lymphocytes. Collectively, these data reveal that irradiation severely impairs HSCs and various progenitors in the BM and spleen; however, these progenitors do not exhibit uniform responses to irradiation.

**Figure 5.**
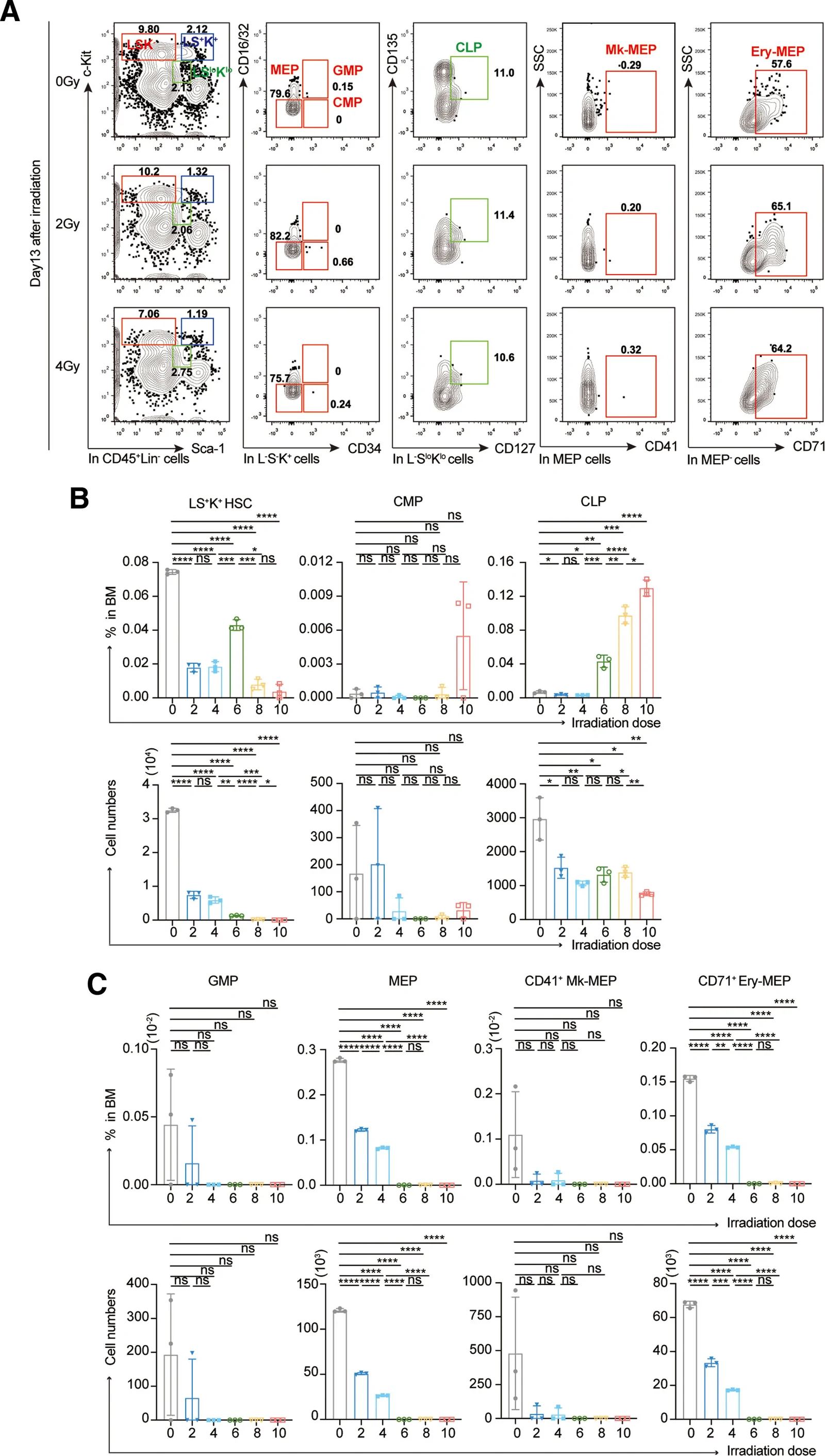
Hematopoietic stem and progenitor cells gradually recovered from irradiation 13 d after irradiation. (A) Representative profiles and (B–C) the percentage and absolute cell numbers of various hematopoietic stem and progenitor cell subsets, including Lin^−^Sca-1^+^c-Kit^+^ (LS^+^K^+^) HSCs, LS^−^K^+^CD34^+^CD16/32^−^ CMPs, LS^lo^K^lo^CD127^+^CD135^+^ CLPs, LS^−^K^+^CD34^+^CD16/32^+^ GMPs, LS^−^K^+^CD34^−^CD16/32^−^ MEPs, CD41^+^ Mk-biased MEPs, and CD71^+^ Ery-biased MEPs 13 days after irradiation (n = 3). CLP = common lymphoid progenitors, CMP = common myeloid progenitors, Ery = erythrocyte, GMP = granulocyte-monocyte progenitors, HSC = hematopoietic stem cell, MEP = megakaryocyte-erythroid progenitors, Mk = megakaryocyte.

At day 13 post-irradiation, the count of BMNCs in the 2 Gy group had returned to normal levels (**Fig. 3A**), while the count of SPNCs was still significantly lower than that in the 0 Gy group (Supplementary Fig. 1A, http://links.lww.com/BS/A106). This observation indicates that the quantity of BMNCs, rather than SPNCs, in the low-dose irradiation group normalized approximately 2 weeks after irradiation. In contrast, at higher doses (≥4 Gy), all irradiated mice exhibited significantly lower counts of both BMNCs and SPNCs until death (**Figs. 1C, 3A**, and Supplementary Fig. 1A, http://links.lww.com/BS/A106). The count of HSCs in both the BM and spleen across all groups remained significantly lower than that of the 0 Gy group (**Fig. 5B** and Supplementary Fig. 3B, http://links.lww.com/BS/A106). However, compared to the status observed 7 days post-irradiation, the loss of HSCs in the BM of the 2 Gy group decreased from 92.1% to 77.1% (**Fig. 4B and 5B**) and from 78.8% to 21.4% in the spleen (Supplementary Figs. 2B and 3B, http://links.lww.com/BS/A106). Additionally, the number of CLPs in the spleen, rather than in the BM, of the 2 Gy group increased to normal levels (*P* = .1251) (**Fig. 5B** and Supplementary Fig. 3B, http://links.lww.com/BS/A106). From the recovery trends of HSCs and CLPs, it appears that the spleen exhibits a better recovery outcome than the BM. Furthermore, at lower doses of exposure (≤4 Gy), the counts of other progenitors, including CMPs, GMPs, MEPs, Mk-MEPs, and Ery-MEPs, in both the BM and spleen increased slightly 13 days following irradiation (**Fig. 5B, C** and Supplementary Fig. 3B, http://links.lww.com/BS/A106). In contrast, at higher doses (≥6 Gy), the majority of these progenitors died within 7 days post-irradiation (**Fig. 4C and 5C** and Supplementary Figs. 2B and 3B, http://links.lww.com/BS/A106). Taken together, these findings suggest that the numbers of BMNCs and SPNCs are dose-dependent and that HSCs and CLPs within these populations exhibit greater resistance to low-dose irradiation compared to other progenitor cells.

### 3.4. TBI impairs the multilineage differentiation capacity of HSPCs in the BM and spleen

**Figure 6.**
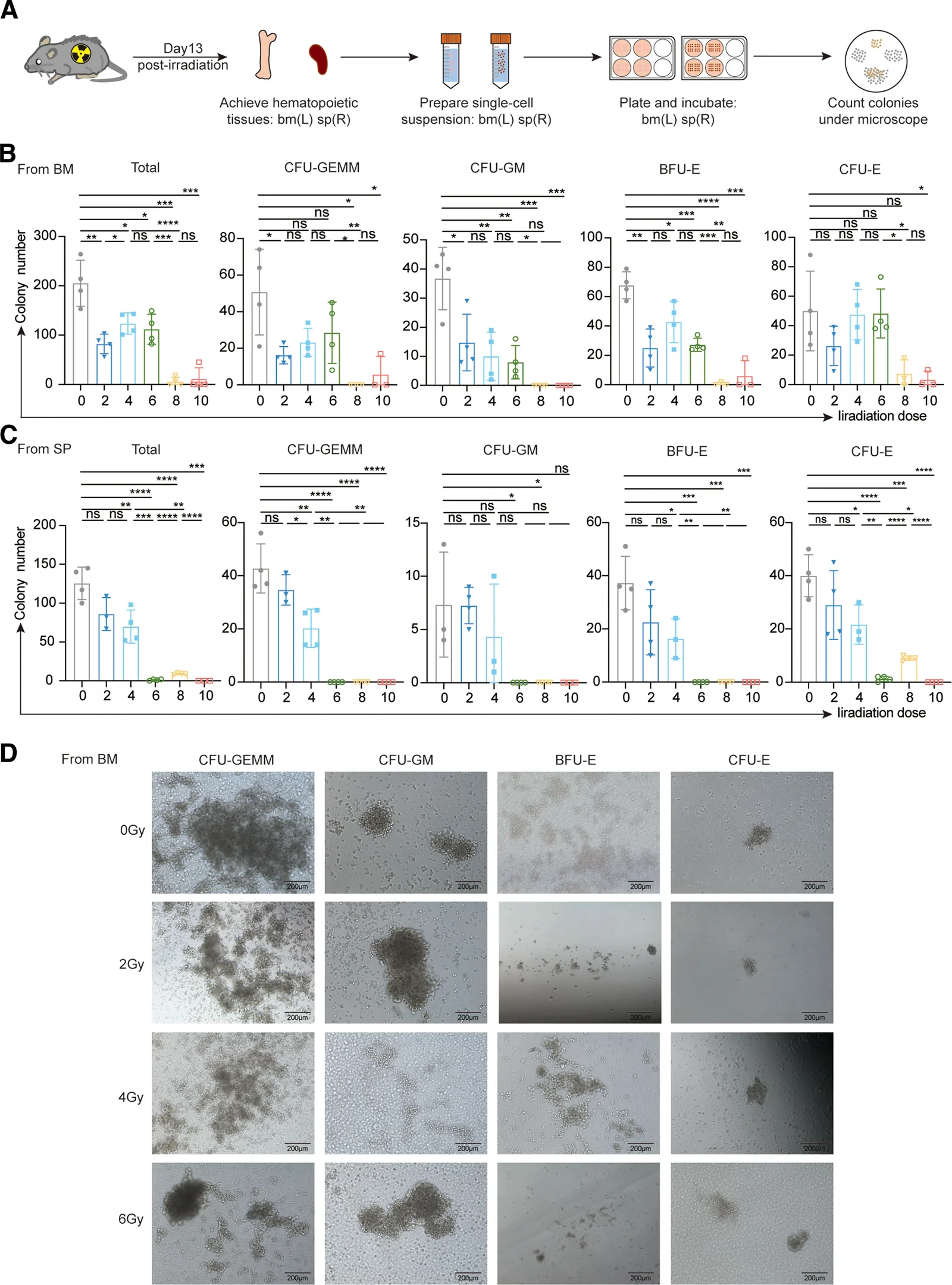
Irradiation impaired the multilineage differentiation capacity of hematopoietic stem and progenitor cells in the bone marrow and spleen 13 d after irradiation. (A) Schematic diagram of the CFU assay. The colonies were derived from (B) bone marrow nucleated cells and (C) spleen nucleated cells at day 13 post-irradiation, following an additional 10 to 12 d of culture in M3434 (n = 3–4). (D) Representative morphological images of CFU colonies from the bone marrow, including BFU-E, CFU-E, CFU-GM, and CFU-GEMM. Scale bar, 200 µm. BFU-E = burst-forming unit-erythroid, BM = bone marrow, CFU = colony-forming unit, CFU-E = colony-forming unit-erythrocyte, CFU-GEMM = colony-forming unit-granulocyte/erythrocyte/macrophage/megakaryocyte, CFU-GM = colony-forming unit-granulocyte/macrophage, SP = spleen.

Our results indicated that the quantities of BMNCs, SPNCs, and HSPCs in the BM and spleen of the mice were restored to varying degrees 13 days post-irradiation (**Fig. 3A** and Supplementary Fig. 1A, http://links.lww.com/BS/A106). However, it remains unclear whether the multilineage differentiation function of HSPCs in these tissues was also restored. To evaluate the recovery of HSPC differentiation function 13 days post-irradiation, equal cell numbers of BMNCs and SPNCs were collected from each group for CFC assays (**Fig. 6A**). These findings revealed a significant reduction in the colony-forming capacity of HSPCs in the BM, irrespective of the irradiation dose; however, the relationship between colony number and irradiation dose did not follow a consistent pattern (**Fig. 6B and D**). Following irradiation with a low dose of 2 Gy, clonal formation of splenic HSPCs remained indistinguishable from normal (**Fig. 6C** and Supplementary Fig. 3C, http://links.lww.com/BS/A106), consistent with our flow cytometry analysis results, which showed that splenic HSPCs in the 2 Gy group exhibited better recovery in cell numbers compared to those in the BM (**Fig. 5B** and Supplementary Fig. 3B, http://links.lww.com/BS/A106). In contrast, at higher doses (≥6 Gy), splenic HSPCs appeared more susceptible to damage from irradiation than their BM counterparts; irradiation doses exceeding 4 Gy completely inhibited the differentiation function of splenic HSCs (**Fig. 6C** and Supplementary Fig. 3C, http://links.lww.com/BS/A106), whereas BM HSPCs retained some differentiation function until the irradiation dose exceeded 6 Gy (**Fig. 6B and D**). These results suggest that HSPCs in the BM and spleen exhibit distinct response patterns to external stimuli.

## 4. DISCUSSION

The hematopoietic system is an organ that can be directly affected by irradiation, whether from radiotherapy or occupational exposure.^18^ Compromised hematopoiesis may result in an inadequate supply of blood cells for the body and can also lead to the production of abnormal blood cells. Therefore, it is essential to study the responses of various hematopoietic cells to irradiation, the effects of different doses of irradiation, and how these responses evolve over time. This knowledge is crucial for radiation prophylaxis in both clinical treatment and occupational settings.

The efficacy of myeloablation through TBI is associated with factors such as dose rate, dose fraction, and the interval between irradiations. In this study, the dose rate was fixed at a lower value of 0.615 Gy/min to facilitate the observation of different cellular responses to irradiation. Fractionated TBI is commonly used in practice due to its reduced irradiation toxicity while maintaining comparable efficiency in killing tumor cells compared to single high-dose irradiation.^14,19^ Consequently, to avoid a significant mortality occurring within a short time frame, mice subjected to 8 or 10 Gy of irradiation were irradiated in fractions of (4 + 4) Gy or (5 + 5) Gy, with an interval of 4 hours between fractions, as this approach has been demonstrated to enhance survival rates and transplantation outcomes.^14^

In the present study, we first examined the peripheral blood of mice, which contains a wealth of hematopoietic information and found that WBCs represent the most severely damaged cell population in the peripheral blood following irradiation (**Fig. 2A**). Low-dose (2 Gy) irradiation significantly reduced the population of peripheral B cells, while higher doses (≥6 Gy) effectively eliminated T cells, whose recovery became increasingly difficult at later stages, ultimately leading to their complete elimination (**Fig. 2C**). T cells are primary contributors to the induction of acute graft-versus-host disease, which remains a major obstacle to successful HCT.^20^ Therefore, high-dose irradiation is essential for enhancing the success rate of HCT. In the context of hematopoietic malignancies, radiotherapy is considered the most effective treatment for lymphoma,^21,22^ and our present study further demonstrates that the cytotoxic effects of irradiation on T, B, NK cells are relatively pronounced. However, their progenitor cells, CLPs, particularly those in the BM, exhibited a different response to irradiation. BM CLPs showed a rapid increase by day 1, resulting in their numbers far exceeding those of the normal group (the 0 Gy group) (**Fig. 3C**). This expansion was temporary, as the number of BM CLPs significantly decreased by day 7 post-irradiation (**Fig. 4B**). Furthermore, the levels of RBC, HGB, PLT, and RET in high-dose (≥4 Gy) groups continuously declined and did not return to normal levels even 13 days following irradiation (**Fig. 2A**). This decline contributed to the severe damage observed in their progenitor cells (**Figs. 3C, 4B, and 5B**), resulting in an inability to replenish functional RBC, HGB, PLT, and RET.

Another application of irradiation is the removal of HSPCs prior to HCT, thereby sparing a niche for the transplanted stem cells. Clinically, TBI is typically combined with chemotherapy as a part of conditioning regimen. In animal studies, TBI is often utilized alone as a myeloablative regimen before transplantation.^23^ Our experimental results indicated that the numbers of various HSPC subsets significantly decreased 1 day after irradiation, reaching their lowest levels after 7 days. Furthermore, recovery of the major HSPC subpopulations only occurred in low-dose groups (≤4 Gy) within approximately 2 weeks post-irradiation, although their levels remained significantly lower than those in the non-irradiated group. Additionally, the results of CFU assays demonstrated that the multilineage differentiation capacities of these restored HSPCs in the BM of the 4 Gy group and the spleen of the 2 Gy group were fully regained. Consequently, the remaining recipient cells in the BM or spleen of the mice did not pose a threat to the newly transplanted stem cells. In previous studies involving mouse xenogeneic or allogeneic cell transplantation, the procedure is typically conducted 4 to 6 hours after irradiation. However, the results of the current study indicated that even at 4 hours post-irradiation, the mice remained in the acute phase, and HSPCs were not completely eliminated. Notably, 7 days after irradiation, both the number and function of HSPCs were significantly impaired. Transplantation at this later time point may thus enhance graft chimerism, ensuring that the newly generated cells more accurately reflect the viability of the donor stem cells.

Transplanted HSPCs migrate to hematopoietic tissues, particularly the BM, as well as extramedullary hematopoietic tissues such as the spleen, where they undergo proliferation and reconstitution following transplantation.^17^ In this study, we observed differences in resistance and response to irradiation between BM HSPCs and splenic HSPCs. Compared to various progenitor cells, including GMPs, CMPs, MEPs, Mk-MEPs, and Ery-MEPs, both BM and splenic HSCs exhibited greater resistance to high-dose irradiation. This increased resistance may be attributed to the fact that the majority of HSCs are quiescent under physiological conditions, which helps prevent stem cell exhaustion; they rely on their committed progeny to generate terminally differentiated cell types across each lineage.^24^ However, the response of CLPs to irradiation differs between the BM and spleen. BM CLPs experienced a temporary increase in cell number 1 day following irradiation, whereas splenic CLPs decreased significantly during this period (**Fig. 3C** and Supplementary Fig. 1C, http://links.lww.com/BS/A106). Additionally, splenic CLPs in the 2 Gy group demonstrated better recovery 13 days following irradiation compared to those in the BM (**Fig. 3C** and Supplementary Fig. 1C, http://links.lww.com/BS/A106). Furthermore, the number of HSCs in the low-dose (2 Gy) group exhibited varying degrees of decline following irradiation: specifically, a 71.5% decline on day 1, a 92.11% decline on day 7, and a 77.1% decline on day 13 in the BM, while in the spleen, there was a 92.9% decline on day 1, a 78.8% decline on day 7, and a 21.42% decline on day 13 (**Figs. 3C, 4B, and 5B**, Supplementary Figs. 1C, 2B, and 3B, http://links.lww.com/BS/A106). These findings suggest that, relative to BM HSCs, splenic HSCs are more severely damaged by irradiation but demonstrate a greater capacity for recovery.

Specific observations of hematopoietic cell responses to radiation provide a fundamental blueprint for radiation prevention and treatment. Different cell types exhibit varying sensitivities to distinct doses of irradiation, serving as a reference for selective prevention strategies. The timing of radiation-induced cellular damage indicates optimal windows for pharmacological intervention, which can yield favorable outcomes. The capacity for spontaneous recovery from irradiation-induced damage reflects the severity of that damage and suggests the extent and duration of pharmacological intervention required to prevent overtreatment. Additionally, this study may offer guidance for the conditioning regimen prior to HCT. By considering the responses of various hematopoietic cell types to irradiation, along with the specific cell types targeted for elimination in the pretreatment plan, an appropriate irradiation intensity can be determined. Moreover, advances in prevention and treatment strategies are closely linked to a deeper understanding of the mechanisms underlying hematopoietic cell responses to radiation. Ionizing radiation primarily induces cellular damage through the generation of free radicals, including reactive oxygen species (ROS) and nitrogen oxides (NOS).^1,2^ Different levels of ROS can lead to varying degrees of DNA oxidative damage, autophagy, apoptosis, cellular senescence, alterations in the cell cycle, functional defects, and epigenetic modifications.^25,26^ This understanding provides a critical entry point for in-depth investigations into the mechanisms governing hematopoietic cell responses to radiation.

## 5. CONCLUSIONS

Our experiments demonstrate that different types of PBCs exhibit varying responses to irradiation. Specifically, B cells show heightened sensitivity to irradiation, while T cells display a dose-dependent response. HSCs exhibit greater tolerance to irradiation compared to most progenitor cells, likely due to their lower activity levels relative to those of progenitor cells. Among these progenitor cells, CLPs show greater resistance to irradiation than CMPs, and their intrinsic stress response may mitigate some of the damage caused by irradiation. Furthermore, significant differences exist in the extent of damage and recovery rates of HSCs following irradiation in intramedullary versus extramedullary hematopoietic tissues. Splenic HSCs experience substantial loss following irradiation but recover more effectively than HSCs in the BM. Additionally, the multilineage differentiation capacity of an equal number of HSPCs that have recovered from injury does not fully return to baseline levels. A thorough understanding of their reactions to irradiation is also valuable for guiding post-irradiation treatment strategies involving damage-repairing drugs.

## ACKNOWLEDGMENTS

This study was supported by grants from the CAMS Innovation Fund for Medical Science (2021-I2M-1-019 to Y.G. and W.Z.), the National Natural Science Foundation of China (92068204 to Y.G., 82370120 to Y.L., and 82200126 to W.Z.), Young Scientific and Technological Talents (Level Two) in Tianjin (QN20230216 to Y.L.), and a SKLEH-Pilot Research Grant.

## Supplementary Material
