# Supplementary material for: The inhibitory impact of various total body irradiation doses on the hematopoietic system of mice
Source: Blood Sci. 2024 Dec 20;7(1):e00214. doi: 10.1097/BS9.0000000000000214 (PMC11666143; doi:10.1097/BS9.0000000000000214)
Supplement: Supplementary file 1 [file bs9-7-e00214-s001.pdf]

Supplementary Figures 1-3

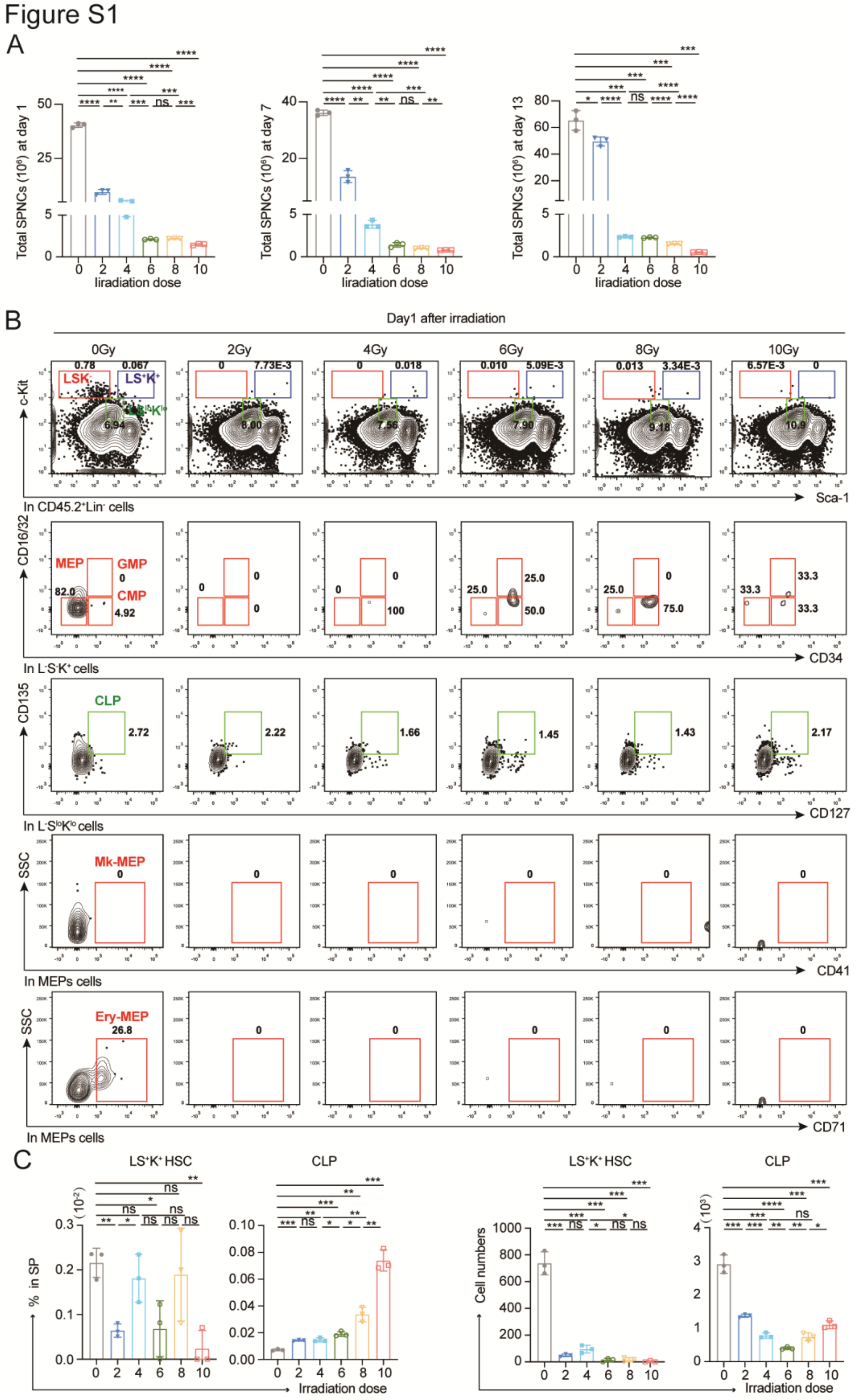

Supplementary Figure 1. Irradiation resulted in significant damage to splenic hematopoietic stem and

progenitor cells one day post-irradiation. (A) Total cell numbers of splenic nucleated cells (SPNCs) at day 1, 7, and 13 post-irradiation (n = 3). (B) Representative profiles and (C) the percentage and absolute cell numbers of various HSPC subsets, including Lin$^-$Sca-1$^+$c-Kit$^+$ (LS$^+$K$^+$) HSCs and LS^{lo}K^{lo} CD127$^+$CD135$^+$ common lymphoid progenitors (CLPs) at day 1 post-irradiation (n=3).

Figure S2

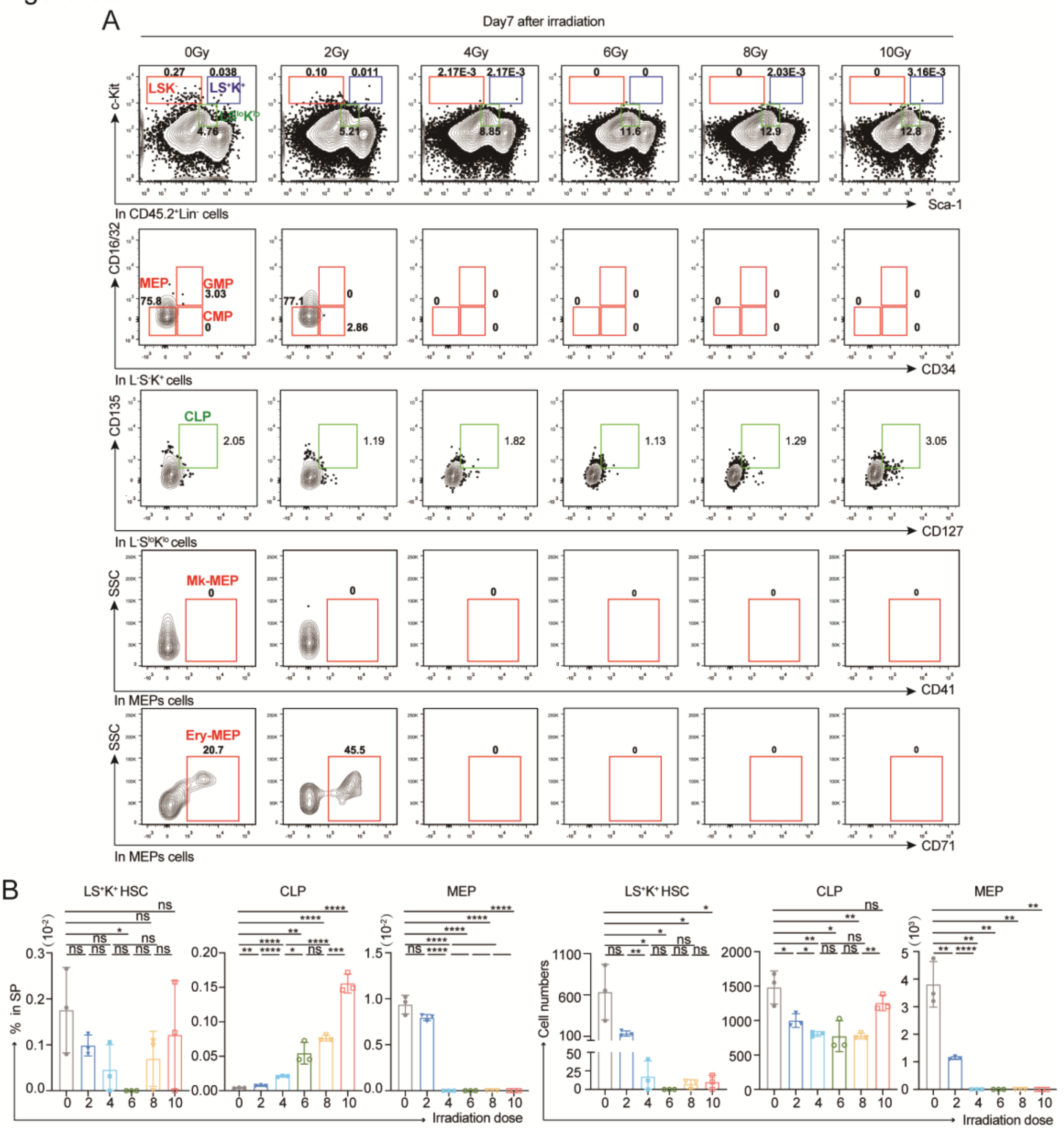

Supplementary Figure 2. The irradiation damage to hematopoietic stem and progenitor cells in the spleen reached its lowest level at day 7 post-irradiation. (A) Representative profiles and (B) the percentage and absolute cell numbers of various HSPC subsets, including Lin$^-$Sca-1$^+$c-Kit$^+$ (LS$^+$K$^+$)

HSCs, LS^{lo}K^{lo} CD127$^+$CD135$^+$ common lymphoid progenitors (CLPs), and LS$^-$K$^+$ CD34$^-$CD16/32$^+$

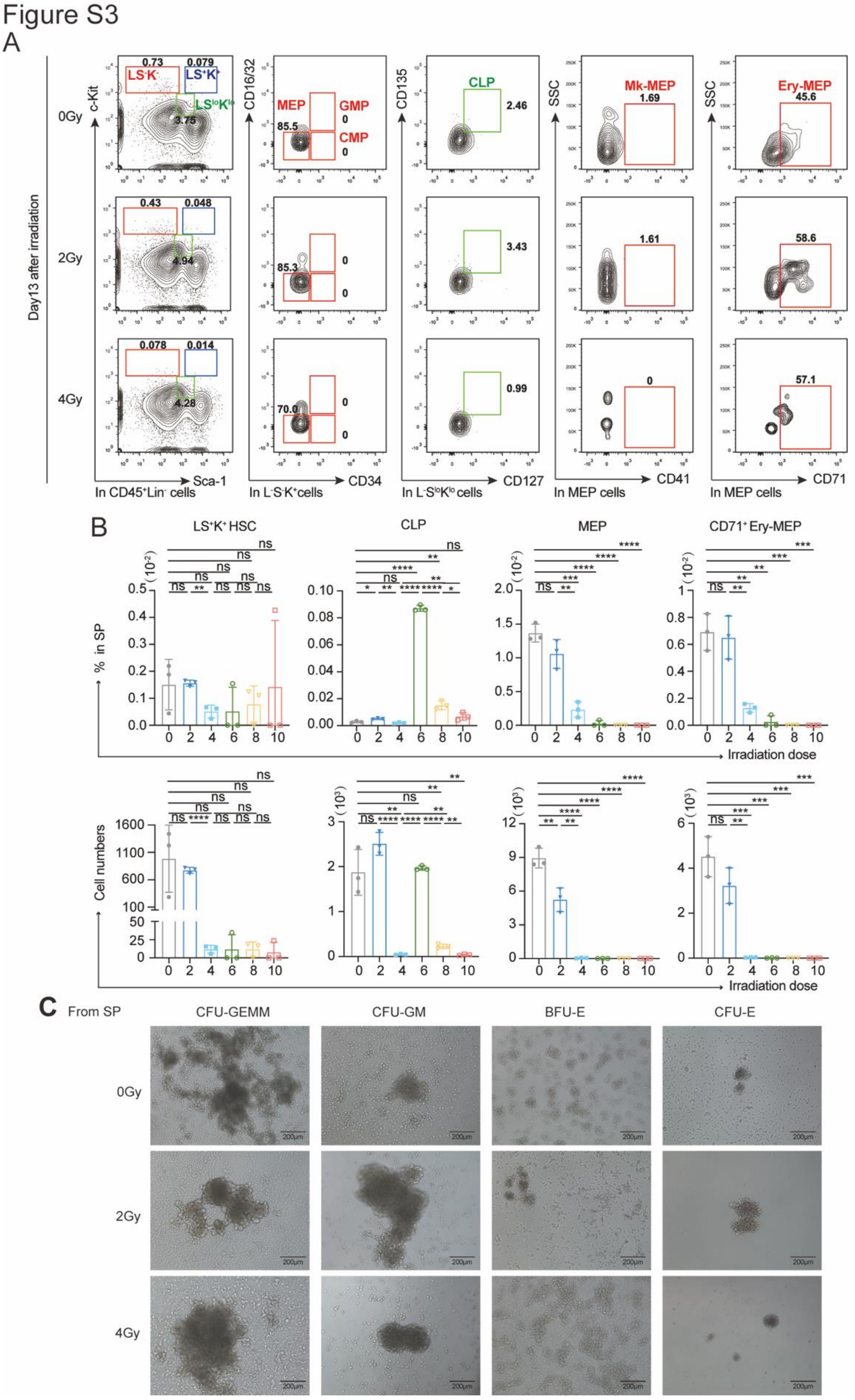

Supplementary Figure 3. Hematopoietic stem and progenitor cells gradually recovered from irradiation

damage 13 days post-irradiation. (A) Representative profiles and (B) the percentage and absolute cell numbers of various hematopoietic stem and progenitor cell subsets, including $Lin^-Sca\text{-}1^+c\text{-}Kit^+$ (LS^+K^+) HSCs, $LS^{lo}K^{lo}$ $CD127^+CD135^+$ common lymphoid progenitors (CLPs), and LS^-K^+ $CD34^-CD16/32^+$ megakaryocyte-erythroid progenitors (MEPs), and $CD71^+$ erythrocyte (Ery)-biased MEPs at day13 after irradiation (n=3). (C) Representative morphological images of CFU colonies from the spleen, including BFU-E, CFU-E, CFU-GM, and CFU-GEMM. Scale bar, 200 μm.
